# Student Moods Before and After Body Expression and Dance Assessments. Gender Perspective

**DOI:** 10.3389/fpsyg.2020.612811

**Published:** 2021-01-08

**Authors:** Mercè Mateu, Silvia Garcías, Luciana Spadafora, Ana Andrés, Eulàlia Febrer

**Affiliations:** ^1^Complex System Research Group, Institut Nacional d’Educació Física de Catalunya, Universitat de Barcelona, Barcelona, Spain; ^2^Collaborator of Motor Action Research Group, Institut Nacional d’Educació Física de Catalunya, Universitat de Lleida, Lleida, Spain; ^3^Institut Nacional d’Educació Física de Catalunya, Universitat de Lleida, Lleida, Spain; ^4^Blanquerna Foundation, Ramón Llull University, Barcelona, Spain; ^5^Conservatori Superior de Música de les Illes Balears, Palma, Spain

**Keywords:** body language, well-being, mood, physical education, assessment, dance

## Abstract

Body expression and dance are activities that contribute to the integral well-being of people. In an educational context, the process of evaluating our students implies variations in their moods. This study tackles the states of mind that students perceive before and after the evaluation of a practice in the subject of *Body expression and dance*, developed through choreographies, that were, previously rehearsed, and later presented to the rest of the class in a specific session. Our main interest was the obtention of information on the states of mind of the students prior to the evaluation of their choreographies (cooperative task), and again once they had been performed. The study design consisted of two phases: two different choreographies separated by an interval of 2 months. The students were asked about their moods before and after performing their choreographies, which were evaluated. The participants, 167 in total, 35 women (20.5%) and 132 men (79.5%), aged 18 to 22 years old, filled out a POMS (Profile of Mood States) questionnaire, before and after the evaluation of each occasion. Differences were found in the stress-anxiety, vigor-activity, fatigue-immobility scales and the total mood score (PGMS). In all of them, except for the fatigue-immobility scale, we found a decrease in scores after the performance, compared to scores before. For the Stress-Anxiety factor, lower values were observed in the post-tests in comparison to the pre-tests, and also lower values in the pre- and post-test scores regarding the second choreography. We conclude that the practical evaluation of the subject through choreography modifies the mood states of the students, favoring their well-being after its execution, which is why evaluation through practice is considered a positive element in the educational process.

## Introduction

Body expression and dance contribute to the emotional well-being of those who practice them, due to the extraordinary cultural diversity of the practices, the interpersonal relationships that they generate, and the intelligent decisions that are made during their practice (Stinson, 2000; Mau, 2015). World dances constitute a cultural heritage of great value to humanity (Goulimaris et al., 2014; Fauntroy et al., 2020).

### Dance, Dances, and Cultural Diversity

Culture in different parts of the world manifests itself through widely varied productions and through different expressive languages: plastic, musical, literary, audiovisual and, among them, also expressions related to human motor skills (Perez and Thomas, 2000). In this sense, human expression is also available through the expression of bodies, games, and dances of different cultures. Each dance form in each country or area of the world shows diverse forms of expression and communication (Stuckey and Nobel, 2010). Human motor skills are enriched with the different forms of relationship with other dancers, with the space used to dance, with the different rhythmic structures, and with the objects that are sometimes used in dances (Mateu, 2010). Dance reveals the poetic, symbolic and referential function of human expressiveness.

In addition to this cultural richness, a specialized literature review helps explaining aspects of physiological and emotional well-being at an individual level (Douka et al., 2019), as well as at a sociological and community level, which are derived from the practice of dance. There are numerous studies on traditional dances, folk dances, jazz dance, among other dance modalities, and the benefits that they bring from an emotional standpoint to all ages, conditions or social statuses, including all kinds of disabilities (Bremer, 2007; Alpert et al., 2009). The dance experience is not only or simply a beneficial physical experience recommended by institutions such as the British Heart Foundation National Center for Physical Activity and Health, but it also offers a way to be sociable, to have fun promoting a spirit of community, and educating in cooperation and peace. It is a language without words, that promotes equal opportunities, with enormous potential as a cooperative activity. Dance is a world heritage, as manifested by organizations such as the International Dance Council, a collaborating entity of UNESCO, the United Nations organization for education, science and culture. Dance is part of local traditions, and it generates its own internal rules of participation.

Currently, there are many projects that aim to preserve the ephemeral heritage of dance. These projects aim to document, archive and therefore preserve the history of dance for future generations. Among them, the non-profit organization Diehl + Ritter is worth mentioning. Recently, it has launched a website^1^ to facilitate access to funds from the German Federal Cultural Foundation for artistic projects dedicated to the cultural heritage of dance. The European Heritage Awards/Europa Nostra Award is likewise worth highlighting, whom, in their research section award outstanding research projects that lead to tangible effects in the conservation and enhancement of cultural heritage in Europe (European Union, 2020). In 2016, Tanzfonds Erbe was one of 28 winners from 16 countries to receive the EU Prize for Cultural Heritage/Europa Nostra Awards in the category Education, Training and Awareness-raising for its achievements in preserving and communicating the cultural heritage of dance.

Also, the project Terpsichore^2^ aims to study, analyze, design, research, train, implement and validate an innovative framework for affordable digitization, modeling, archiving, e-preservation and presentation of Intangible Cultural Heritage (ICH) content related to folk dances, in a wide range of users as dance professionals, dance teachers, creative industries and general public (Doulamis et al., 2017; Rallis et al., 2018). This intangible (ICH) can be extended to other types of dance: classical, contemporary, and urban, among others, which also constitute a heritage of great value in the history of humanity. Our study is developed in relation to creative dance along university students who use this resource as a form of individual and collective expression.

### Internal Logic of Body Expression and Dance Activities

Many authors place motor expression practices among the domains of motor action, which were labeled as Artistic Physical Activities by Parlebas (2001). We define them as cooperative psychomotor or sociomotor practices in which a praxis and a symbolic dimension are combined (Mateu, 2010; Mateu and Bortoleto, 2011; Torrents et al., 2011; Mateu and Torrents, 2012; Mateu and Lavega, 2017).

Through their internal logic (Parlebas, 1988, 2001, 2017), we observe different possibilities for interpersonal relationships. In this sense, the contribution of authors such as Urdangarín (2009), on Basque dances, or Mateu (2010) on collective dances, are worth highlighting, specially from the perspective afforded by Professor Pierre Parlebas regarding the science of motor action.

Urdangarín (2009) states that, for example, Basque dances are social activities and that, in this sense, they do not only establish a relationship with non-dancers or with their audience, but they also build a relationship for the dancers themselves (in their compositional demands such as meeting in pairs, the type of motor interaction that is requested, or the adoption of sociomotor roles). An analysis of the different ways in which dancers have to collaborate with each other, through body contact or in the use of objects is specially interesting. In relation to the interaction with other dancers, Mateu (2010) explains that one can find dances with contact or without contact, psychomotor, commotor, or sociomotor dances, and/or with a invariable partner or with a changing partner.

There are also studies on the internal logic (Parlebas, 2001) of the different types of dances, in relation to collective dances (Larraz, 1989; Plana, 1993), contemporary dance (Guerber Walsh et al., 1991; Comandé, 2002), classical dance (Troya, 2015), Spanish dance (teachers at the Professional Dance Conservatory of the Institut del Teatre de Barcelona), capoeira (Jaqueira and Araujo, 2013; Ríos, 2015), or urban dances (Bérillon and Ramires, 2014). In all of them, the different forms of interpersonal relationships that can be established stand out, in addition to the various forms of relationships with the space, time, and objects involved.

In addition, in recent years, some studies have delved into the moods and emotions generated by these interrelationships in cooperative motor-expression practices and, in our case, through the experiences of university students (Romero-Martín et al., 2015, 2017). Sáez de Ocáriz et al. (2014) studied the emotional experience that the practice of expressive motor situations of cooperation provokes in university students in Sciences of Physical Activity and Sports. Lavega et al. (2017), also explored the effect of gender and group gender composition in emotional experiences of men and women when participating in different individual and cooperative games. In a study by Romero-Martín et al. (2017), the effects of three types of psychomotor practices were analyzed, that is, not including motor interaction (motor games, motor expression and motor introjection) on the state of positive, negative and ambiguous emotions in women and men.

### Body Expression and Dance in the Physical Education Curriculum

The Spanish Physical Education curricula (Generalitat de Catalunya, 2015; Ministerio de Educación y Ciencia, 2015) incorporates a “contents block” dedicated to Expression-Communication. Among the objectives of compulsory secondary education in Spain, one finds the goal to: «appreciate artistic creation and understand the language of different artistic manifestations, and use various means of expression and representation» (Boletín Oficial del Estado (BOE), Ministeri d’Educació, Cultura i Esport, 2015:9). The titles for these block of contents vary depending on their focus on diverse activities at a national level: *Expression and Dance, Body Expression, Dance and Circus, Physical or Body Expression Activities, Artistic Activities*, among others; or, for example, *Communication and body expression* in Catalonia (Generalitat de Catalunya, 2015). Each one of these meanings responds to the work on the dimension of body communication and expression.

One of the elements of the curriculum is evaluation. The evaluation is layed out in relation to the objectives, contents, evaluation criteria and basic competences of the subject. The evaluation process is characterized by: collecting information, analyzing this information and making judgments, making decisions in accordance with the judgment issued, related to two types of purposes: aimed at regulating the difficulties and errors that arise throughout a process of teaching-learning and, related to the assessment of the results of a teaching-learning process (Sanmartí, 2007, 2010).

Regarding the pedagogical moment in the evaluation of the students, most studies coincide in underlying the importance of developing it as an additional educational moment, within the whole pedagogic process. A source of concern for us was found in the process of obtaining information based on the emotional management of our students, given that some of them might experience it negatively. In this sense, we aimed to understand the educational meaning that this moment could afford. As Ruffin (2016) points out, the presentation of a collective performance in front of others classmates constitutes a strong emotional commitment. Presenting one’s choreography in public means establishing a privileged connection with fellow spectators, to transport them to an imaginary world. The challenge for each student is based on maintaining this connection despite of their own emotions, risk perception, and presence unforeseen events.

In Physical Education, most of the times, all practices are the object of an evaluation, taking into account the performance and the level of mastery of the activity as shown by the students. But body language and dance represent areas of discovery and experimentation of emotions unknown to the participants. These activities can be the basis of work on symbolic expression and the communication of emotions (Debois et al., 2007). Artistic activities can be an opportunity to become aware of the emotions felt in front of others: for example, during the presentation of a choreographic sequence, fear and, pride of being in front of one’s classmates arise, and develops along the presentation in front of teachers and their judgments.

Within the Body Expression and Communication contents block, and within the section referred to the evaluation of the activity, most educational syllabi contains practical evaluative proposals (Ortíz, 2002; Romero, 2015) under various designations (choreography, scene, representation, creation, product, performance, among others). In 1984, Baffalio-Délacroix, Orssaud-Flamand proposed the coupling of *expression as process and/or expression as result*, inviting an analysis of the pedagogical consequences of a subject such as Physical Education, including progressive sessions without finalist presentations, or rather the educational nature that can be assigned to the choreographic representations during certain moments of the evaluation process.

The different subject syllabi in Spanish Faculties of Physical Activity and Sports Sciences, as well as the teaching programs for subjects in Compulsory Education and Baccalaureate centers, usually incorporate choreographic performances in their student assessments. In our study, a group choreography is performed at a certain moment of the course, and another group choreography when the course is ending, both using a cooperative methodology, and within the qualifying evaluation of the subject of Body Expression and Dance.

### Mood States in the Evaluation of Students

For the purpose of this study, we define “mood state” as a series of feelings, ephemeral in nature, that vary in intensity and duration, and which generally involve more than one emotion (Lane and Terry, 2013). Some authors have illustrated how they perform their subject assessment for “emotional practices” (Hargreaves, 1998; Steinberg, 2008). However, in the literature regarding evaluation and emotion, it is generally the teachers’ emotions that are discussed. This shows that further research is needed for a better understanding of the ways in which students’ evaluations can be interpreted as an “emotional practice.”

Some literature also refers to the mood modifications caused by viewers attending dance and circus shows, and explores some of the components of the experience, along the effects that they could have on attendees and performers both at physical and emotional levels (Rueda et al., 2017). This is also the case of some studies in art education, that focus on esthetic emotions and their assessment in behalf of art students, undergraduate and postgraduate students (Mohammad, 2018).

However, we have not been able to find any research on the emotions that students experience during subject assessment. A qualitative study sought out the moods of students who studied at the Royal Academy of Dance, through the administration of a POMS (Profile of Mood States) questionnaires before and after the students’ performative evaluation (Tedesco et al., 2017). In this particular study, the authors underline the need for further research in order to improve any approaches to subjective states involving practice, previous dance training, and performance evaluation, since they all have been neglected in terms of academic research.

Another interesting aspect that affects students’ moods when performing body language and dance tasks is whether or not they are observed, and by whom. The emotional affectation varies depending on whether the “spectator” is a pair of students, half of the class-group, the entire class-group, or if it is an external públic. Likewise, it varies depending on it being a task with beforehand preparation or an improvisation task (Canales-Lacruz, 2011).

In order to be able to speak properly about the educational contribution of this unique moment, both regarding individual and the group’s pedagogical processes, we asked ourselves which were the states of mind of the students who had to carry them out. We also wondered if there were variations before the staging of the choreographies and after their completion, aiming to detect any possible differences between genres, while paying special attention to how the moods of the participants evolved during the time between scenes. The proposed evaluation activity (group choreography) therefore constitutes a cooperative sociomotor task, which is carried out in front of spectators-classmates and the teachers of the subject.

We understand the incorporation of each scene/choreography at specific moments in the development of the subject as a moment belonging to the pedagogical process of each individual and group of students. In this sense, these scenes/choreographies represent an “especially educational moment.” From the point of view of the teachers, who know and recognize the moods of their students in each moment, this allows a more intelligent management of the student’s evaluation, in the broad sense of the term.

### Dance and Gender Perspective

In previous studies on the experience of emotions in cooperation-opposition games, carried out with in elementary school students, the causes that originated the experience of positive emotions are underlined. In the comments, the boys mainly referred to the winning in the game to justify their emotional experience, while the girls, in addition to this aspect, simultaneously relied on contextual factors such as having fun or laughing during the motor practice. The girls highlighted the motor relationship between developed among peers, the need to cooperate in a group and not feel rejected, mainly by male participants (Alcaraz et al., 2017). Likewise, Muñoz Arroyave et al. (2017) conclude that negative moods are more present in solo games, without competition, as they are practiced by men, and are organized in separate groups.

A study by Gelpi et al. (2014) focused on identifying whether the motor tasks in expression-dance of a psychomotor and cooperative nature elicited the same tendencies of emotional experience among students, examining whether there were differences in emotional intensity based on gender. No significant differences were found in emotional intensity between men and women. The aforementioned study by Lavega et al. (2017) focused on exploring the effect of gender and group gender composition on the emotional experiences of men and women when participating in different individual and cooperative games. In a study by Romero-Martín et al. (2017), in regards to gender, men registered more intense emotional values than women in motor games, as well as in negative and ambiguous emotions, while they had a similar emotional behavior in training practices. expression and introjection.

Working in dance in pairs or mixed groups is an opportunity for both boys and girls to experience feelings of joy and not of confrontation, of comparison with each other. This is also a way to bring out their moods, to find their own authenticity (Debois et al., 2007).

### Aims

The aims of the study are the following:

(a) To obtain information about the pre-mood and post-mood states, before and after performing two group choreographies based on body expression, before a group of classmates and teachers.
(b) To verify if there are differences between the moods prior to a first group choreographic performance (phase 1), and the moods prior to a subsequent group choreography performance (phase 2), after an interval of 2 months.
(c) To analyze the possible differences between men and women in their mood expression before and after performing a choreography of body expression and dance.
(d) To relate the moods shown after the choreography with the explanation of the “desire to show the scene,” and the “sense of security” shown before the evaluation.

The effects of three independent variables were studied:

(a) The beginning and the end (pre-test before the choreographies, and post-test after the choreographies).
(b) The adaptation to the evaluation (the pre-tests of both choreographies and the post-tests of both choreographies).
(c) The gender (female, male),on the intensity of the dependent variables corresponding to the five mood states (MS): ^Negative^ MS: stress-anxiety, depression-dejection, rage-hostility, fatigue-immobility, and ^Positive^MS: vigor-activity.

Our initial hypotheses are:

(1) The moods of the students before an evaluation through motor choreography will be emotionally different from the moods once the performance is over.
  Previous studies have shown that there is modification in the moods of students when they are evaluated since evaluation is an emotional practice (Steinberg, 2008; Tedesco et al., 2017).
(2) The performance that takes place 2 months after the first constitutes a training, so that the intensity of the moods compared to the first will show lower values, Evaluation is part of the process and, as an emotional practice, it can be “trained” (Baffalio-Délacroix and Orssaud-Flamand, 1984; Steinberg, 2008).
(3) The moods before and after the scene will have similar values in men and women.
  Some previous studies show that there are no differences between men and women in the emotional intensity of cooperative activities from the domain of artistic activities (Gelpi et al., 2014; Romero-Martín et al., 2017).

## Methodology

**FIGURE 1:**
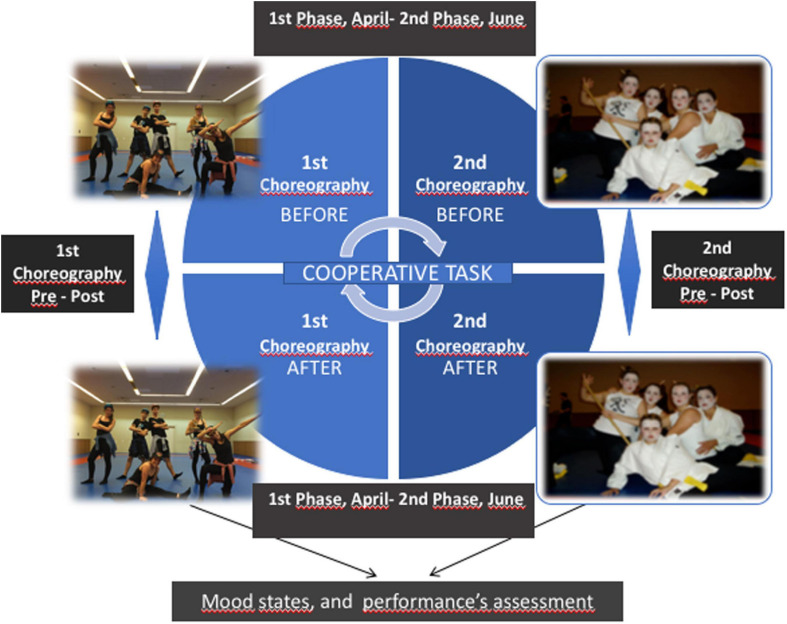
Methodological design.

Two phases were distinguished in this project: the application of the POMS (Profile of Mood States) questionnaire before and after the choreographies of the first scene of body expression and dance in April 2016; and the application of the same questionnaire before and after the completion of the choreographies of the second choreography of body expression and dance in June of the same year (see Figure 1).

The first choreography was performed in groups of six-eight people in a preselected space, with a homogeneous musical background, and it did not include the use of any objects. In the second scene, the number of members of the group was free (from 1 to 40 people – all the members of the class –), and included the choice of performative space, musical background, and optional use of any objects. In both choreographies, previous rehearsals were planned, as to avoid improvisation at the time of the performance. The formation of groups was carried out by association by the students themselves, both regarding the constitution of the groups for the evaluation of the first choreography (limited to 6-8 people) and for the second (free).

### Ethics Statement

Written informed consent was obtained from the individual(s) for the publication of any potentially identifiable images or data included in this article.

### Participants

A total of 166 university students of the Degree in Physical Activity and Sports Sciences from Institut Nacional d’Educació Física de Catalunya at the Universitat de Barcelona, with an initiation level in dance, participated in this pedagogical experience. The sample included 35 women (20.5%) and 132 men (79.5%), aged 20 to 22 years old, *SD* = 1,48.

### Instrument

The Profile of Mood States scale (POMS by McNair et al., 1971) validated in Spanish by Balaguer et al. (1993) was chosen for the applied evaluation of the students’ mood. The pedagogical experience presented in this work was included in the academic program of the subject Body expression and dance, for which the use of a reduced version of the POMS was agreed. The abbreviated form of 29 items (Fuentes et al., 1995) explains 94% of the total variance in the adaptation of the POMS made up of 51 adjectives. In this version, the participants report on their own mood state in relation to the items in the instrument The scale reports on five mood states: stress, depression, anger, vigor, and fatigue. The scale is highly reliable in terms of internal consistency (Cronbach’s alpha of: stress = 0.83, anger = 0.85, vigor = 0.83, fatigue = 0.00.82, and depression = 0.78). The items were assigned a value from 0 (not at all) to 4 (very much), and were grouped into five factors: stress-anxiety, depression-dejection, rage-hostility, vigor-activity and fatigue- immobility. In addition, a global score of mood (PGMS) is obtained. This score is acquired by adding the values of the four factors that are directly related such as tension, depression, hostility, fatigue, and subtracting vigor.

The description of each of these moods, their description, along their number of items and their statement (short version of 29 items), is as follows:

Stress-Anxiety: it is a continuous feeling of physical or emotional stiffness. It can come from any situation or thought that makes one feel frustrated, angry or nervous (Sandín, 2003). Tension is defined by adjectives that reflect increases in musculoskeletal tension. In the short versión here are 6 items that compose it: uneasy, restless (observable psychomotor manifestations), agitated, nervous, tense (that reflect states of diffuse anxiety), and relaxed. Depression-Dejection: persistent feeling of sadness and loss of interest (Lane and Terry, 2013). It constitutes a depressive state, accompanied by a feeling of personal inadequacy. It consists of 5 items: helpless, hopeless, depressed, wretched, sad. Rage-Hostility: in this case, the scales show a state of anger and antipathy directed toward others. It consists of 8 items: furious, annoyed, fighter, irritable, embittered, bitter, angry, and short-tempered. Vigor-Activity: persistent mental and physical activation, predisposition to invest effort and persistence, even during difficulties (Medrano et al., 2015). It is composed of 5 adjectives that suggest a state of mind of vigor, euphoria and high energy: energetic, full of energy, animated, vigorous and active. Fatigue-Immobility: it is a state of chronic physical and psychological exhaustion, the result of an excess of personal demands and continuous mental stress. It is the feeling of being emotionally exhausted and exhausted, due to the situations that surround us (Pedraz-Petrozzi, 2018). It corresponds to a state of dejection (wear), inertia and low energy levels. The following 5 items refer to it: surrendered - exhausted-, inattentive- apathetic-, tired, exhausted and fatigued.

Likewise, the participants were asked dichotomously (Yes-No) about their “desire to carry out the performance” and the “feeling of security” regarding the performance for each scene.

### Process

Initially, a training session was held to explain the procedure and to explain the POMS questionnaire. Some of the competences to be acquired in the subject of Body expression and dance were evaluated through the presentation of two different choreographies to their peers of the rest of the group-class. The choreographies were performed in groups with a time difference of 2 months between them. It was therefore a matter of socio-motor cooperation tasks. The groups included 6 to 8 people, and therefore between 20 and 22 choreographies were performed in each one of the phases. For the students, the first of the choreographies (first phase of the study during April) was the first time in which they performed in front of the rest of their classmates. It was therefore his first public performance. The second choreography in front of their companions, was presented 2 months later (second phase of the study in June). The POMS questionnaire was administered in each of the two phases of the study (months of April and June), before and after the performance of the choreography in front of the rest of the class.

Once the group of 40-42 students had been prepared to perform their respective choreographies in small subgroups, they completed the questionnaire (pre-test) fifteen minutes before starting the evaluation session itself. After performing their choreographies, the 40-42 students spent fifteen minutes filling in the questionnaire again (post-test). The questionnaires were completed by all members of the class (166 students), organized into subgroups of about 40 students (four successive groups). Between 20 and 22 choreographies were performed and evaluated in April, and 20-22 new choreographies 2 months later, in June. It should be noted that the students completed the POMS questionnaire for the post-test without knowing the result of the evaluation of their choreography by the teachers. The conditions necessary for the participants to complete the questionnaires were guaranteed at all times. They also signed an informed consent document.

In addition to filling in the POMS questionnaire, two more questions were asked to complete the information on how they faced the evaluation that they were going to undergo, through the interpretation of it choreographies: (a) Do you feel like performing the choreography? And (b) do you feel safe or secure when performing the choreography? These questions were answered dichotomously with a yes or no.

### Data Analyses

The analyses were carried out using SPSS statistical program, in its 23rd version. A descriptive analyses of the different variables was carried out in terms of means, standard deviations and percentages. Non-parametric Wilcoxon signed-rank test was applied in order to assess the possible differences between the scores obtained on the POMS scales before and after the representation of the dance scenes, as well as to test the possible differences of means between the scores obtained in the two phases of the study (April and June). Likewise, Mann-Whitney U was applied to analyze the possible differences between men and women on the POMS scales. Finally, the relationship between various dichotomous variables was analyzed using chi-square.

## Results

This section presents the results obtained in the analysis of the states gathered before and after the two choreographies of body expression were performed, in the two phases of the study (Phase 1 1st choreography, April, and Phase 2 2nd choreography, June).

We have chosen to pursue a description of the results based on the effect on the different mood factors (dependent variable) of each of the independent variables:

(a) the before-after of each one the choreographies (start or pre-test before the choreography, and end or post-test after the choreography);
(b) the adaptation to the assessment: the pre and post of the first choreography (Phase 1 in the month of April), and the pre and post of the second choreography (Phase 2 in the month of June); and,
(c) gender (feminine, masculine).

Given that the POMS questionnaire identifies 5 factors, the results referring to negatively oriented moods are first described.

(a) Effects of the independent variable Before-After, on ^Negative^MS (SA, DD, RH, FI) and on ^Positive^MS (VA).

In first phase, the mean difference analysis shows that there are statistically significant differences in various items on the POMS scales, as pictured in Table 1. More specifically, differences were found in the stress-anxiety, vigor scale-activity, fatigue-immobility and the total mood score (PGMS). In all of them, except for the fatigue-immobility scale, there was a decrease in the scores obtained after the scene compared to the scores before the scene. However, it should be noted that no statistically significant differences (*p* > 0.05) were found on the depression-dejection and rage-hostility scales between before and after the choreography representations. There is also a decrease in the vigor factor in the post-test.

**TABLE 1:** Scores obtained on the POMS scales before and after the choreography, in the 1st phase.

	Before M (SD)	After M (SD)	*Z*	*p*
Stress-Anxiety (SA)	9.87 (3.77)	5.42 (3.17)	−9.885	<0.0001
Depression-Dejection (DD)	0.97 (1.61)	0.78 (1.89)	−1.925	0.054
Rage-Hostility (RH)	4.15 (2.66)	3.82 (3.25)	−1.729	0.084
Vigor-Activity (VA)	14.41 (3.98)	12.60 (5.24)	−4.127	<0.0001
Fatigue-Immobility (FI)	2.88 (3.15)	4.56 (4.37)	−4.582	<0.0001
Mood score (PGMS)	103.47 (8.34)	101.97 (9.59)	−2.459	0.014

In the second phase of the study, the possible differences in the POMS scales between the moment before and after the representation of the scene (choreography) were analyzed again. In this case, statistically significant differences were found in all scales, as well as in their total (PGMS), producing a decrease in all of them except for the scores obtained on the fatigue-immobility scale, in which the scores obtained after the scene were superior to those obtained before it (see Table 2). We observed a decrease in the vigor factor in the post-test indicating a loss in physical energy and disposition, probably due to physical fatigue.

**TABLE 2:** Scores obtained on the POMS scales before and after the choreography, in the 2nd phase (2 months later).

	Before M (SD)	After M (SD)	*Z*	*p*
Stress-Anxiety (SA)	9.25 (4.39)	5.40 (3.46)	−9.072	<0.0001
Depression-Dejection(DD)	1.50 (2.28)	1.12 (2.61)	−2,560	0.010
Rage-Hostility (RH)	5.04 (3.70)	4.11 (3.90)	−3.993	<0.0001
Vigor-Activity (VA)	14.40 (4.10)	13.39 (4.91)	−2.270	0.023
Fatigue-Immobility (FI)	2.91 (3.31)	5.06 (4.73)	−5.098	<0.0001
Mood state (PGMS)	104.29 (11.23)	102.29 (11.05)	−2.629	0.009

**FIGURE 2:**
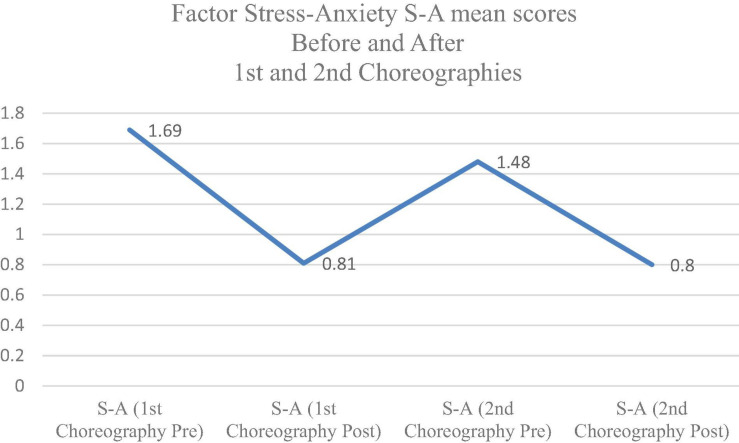
Factor Stress-Anxiety (S-A) mean scores before (pre) and after (post) 1st and 2nd choreography.

Stress-Anxiety is the only one of the negative factors that descends in the second choreography both (see Figure 2) before the execution of the choreography (pre) and after the choreography (post).

(b) Effects of the independent variable Adaptation to assessment, Phase 1 (April) and Phase 2 (June), ^Negative^MS (stress-anxiety, depression-dejection, rage-hostility, fatigue-immobility) and on ^Positive^MS (vigor-activity).

**FIGURE 3:**
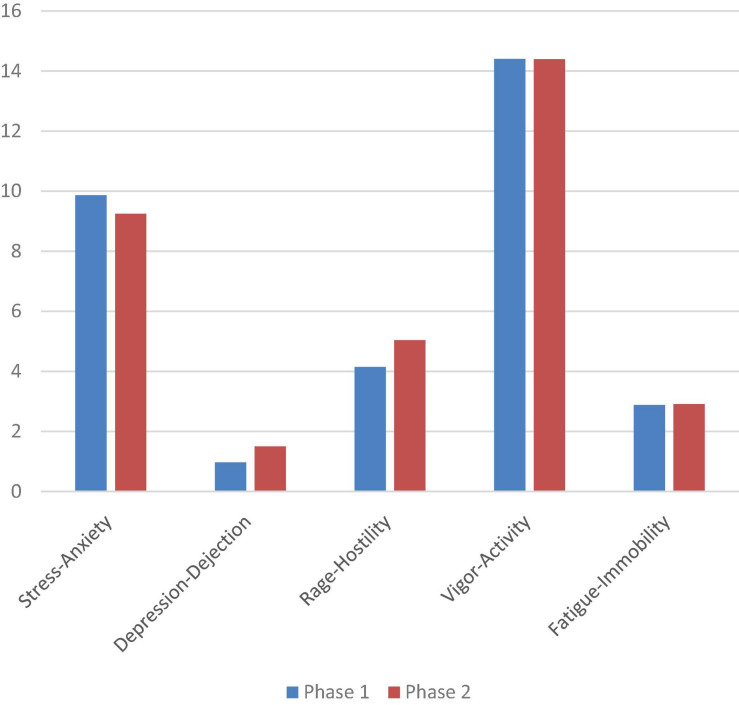
Scores obtained on the POMS scales before the choreography, in phases 1 and 2 (2-month interval): stress-anxiety; depression-dejection; rage-hostility; vigor-activity; fatigue-immobility.

With the aim of analyzing the mood of the participants before the dance and body expression choreographies, a comparison analysis of means between the scores obtained between phases 1 and 2 was performed, considering only the mood states reported before the performance. The analyses show that there are statistically significant differences between the two phases on the stress-anxiety scales (*Z* = -2.418, *p* < 0.05), depression-dejection (*Z* = -2.920, *p* < 0.01) and rage -hostility (*Z* = -2.780, *p* < 0.01). Scores decreased in phase 2 compared to phase 1 on the stress-anxiety scale, but increased on the depression-dejection and rage-hostility scales, as can be seen in Figure 3.

**FIGURE 4:**
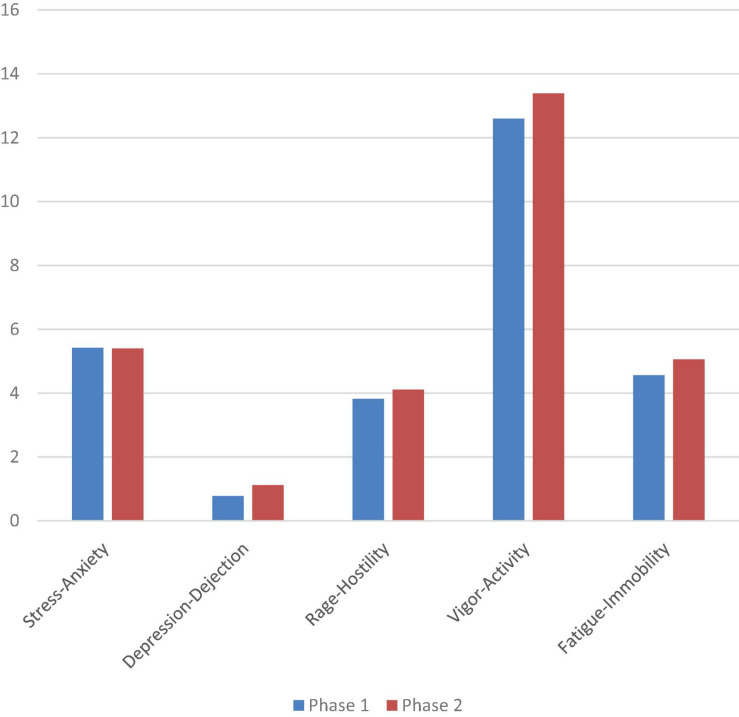
Scores obtained on the POMS scales after the choreography, in phases 1 and 2 (2-month interval between the two): stress-anxiety; depression-dejection; rage-hostility; vigor-activity; fatigue-immobility.

Likewise, the possible differences between phases 1 and 2 were analyzed in terms of the emotions reported after the scene was performed. In this case, no statistically significant differences were obtained in any of the POMS scales or in the global PGMS index between both moments (see Figure 4).

Regarding the ^Positive^Mood, in the Vigor-activity scale in the first phase the post-value (14.40) is practically the same as the pre-value (14.41). And in the second phase, the post-value increases (13.39) compared to the pre (12.6) possibly due to the fact that the groups had intervened in a more creative way in the preparation of their choreography, which in this sense was more original and proper, and remained both mentally and physically activated even after completion.

(c) Effects of the independent variable Gender on ^Negative^MS and ^Positive^MSIn order to analyze a possible gender-related effect on the different mood scales, the scores obtained from men and women were compared. The only differences related to gender were found in the depression-dejection scale in the first phase and before the representation, being the men (*M* = 1.07, *SD* = 1.72) the ones to show higher scores, compared to women (*M* = 0.58, *SD* = 1.00; *U* = 1533.50, *p* < 0.05). No differences were found between men and women in any of the other scales or times.

Similarly, in relation to negative mood states, no differences were found between men and women on the Vigor-Activity scale.

### Relationship of Mood and Complementary Questions

At the same time, the possible association between the “desire to carry out the performance,” the “feeling of security” before the performance of each scene, and satisfaction for each of the moments of evaluation was analyzed.

Considering the results obtained in phase 1, no association was found between the “desire to carry out the performance” prior to the performance with the satisfaction of the participants after the performance. However, greater “safety before representation” was significantly associated with greater satisfaction after representation (*p* < 0.05).

Regarding the results obtained in phase 2, it should be noted that a statistically significant association was found between “the desire before the performance” and the satisfaction after it, both individually (*p* < 0.05) and group (*p* < 0.05). In the same way, in phase 1, a greater “security” before the scene was related to a greater satisfaction after (*p* < 0.0001).

Finally, the comparison of the scores obtained in phases 1 and 2 showed that there was indeed an association between the “desire to carry out the scene” in both time points (*p* < 0.05), the “feeling of security” in the performance of each scene (*p* < 0.0001), and individual satisfaction (*p* < 0.05).

## Discussion

This research examined the effect of evaluation through of two choreographies, performed at the beginning and at the end of two sessions, developed in two different moments in time, and also according to gender, regarding the moods of university students. Some significant differences were observed according to the variables studied. Regarding our initial hypotheses: (1) the moods of the students before an evaluation through motor choreography will be emotionally different from the moods once the performance is over; (2) the performance that takes place 2 months after the first constitutes a training session, so the intensity of the moods compared to the first one will show lower values; and (3) the moods before and after the scene will have similar values in men and women. We can state the following:

The first hypothesis is confirmed, and we can affirm that, even inserted in an evaluation context, the assessment through choreography acts favorably on the perception of positive moods. They act in reducing values such as depression and anger, which demonstrates their potential to positively interact on the students’ mood regarding body language and dance. These results confirm the findings obtained by other studies in which it is found that in relation to emotions, regardless of the type of motor practice that is pursued, positive emotions are perceived with greater intensity than negative or ambiguous ones; especially in cooperative games (Martínez and Valero, 2018). Likewise, the practice of communicative tasks has triggered expressions related to the positive emotion of love, and the use of objects used in some choreographies, seems to facilitate a relaxed environment and humor (Torrents et al., 2011). It also coincides with the statement that cooperative expressive motor practices arouse intense positive emotions in students that allow the development of social skills and abilities to improve school coexistence (Sáez de Ocáriz et al., 2014). This is also reproduced in the study by Miralles et al. (2017), in which it has also been established that in a non-competitive situation, the intensity of our emotions is very high as well, especially regarding positive emotions.

Relating to others generates a greater intensity of emotions, especially when we relating from cooperation. Likewise, and even in the case of physical education and sport students, and not dance students, we find a great parallel with the results found in their research with dance students from the Royal Ballet, with appliance the POMS questionnaire (Tedesco et al., 2017). Vigorous activity is one of the mood factors evaluated by the POMS, which suggests synonyms for animation, energy, or activity. A decrease in the vigor factor was also found among these students in the post-test, indicating a loss of physical energy and disposition, probably due to physical fatigue, and with an increase in the fatigue-immobility factor. Regarding possible negative moods and/or emotions, the literature suggests that sport and dance improve subjective well-being but identify negative feelings of competence and ability (Mansfield et al., 2018). The quantity and quality of published evidence on dance and sports interventions to improve subjective well-being is little.

The second hypothesis is also confirmed, especially in relation to the stress-anxiety factor that decreases in the second phase (choreography evaluated in June), compared to the first phase (choreography evaluated in April). The values of the stress-anxiety factor decrease both before and after the evaluation. This leads us to think that there has been an adaptation, a training of this factor, which leads to a decrease in the stress-anxiety factor in this second evaluation. This also coincides with Tedesco et al. (2017), when explaining that stress is associated with a situation prior to the evaluation, and is related to expectations of performance, competence and fear of not being prepared. This statement coincides with the responses of our students in their desire to perform the choreography, and the greater or lesser degree of security that they felt while performing it. The tension, according to Rebustini and Machado (2012), points toward a clear downward trend in the value after the activity. It should also be noted that in our study, the vigor-activity factor does increase in the second phase compared to the first, possibly due to a greater participation in the creation of the choreography by the students, compared to the first choreography that was more limited by teachers. This sense of authorship and even a certain empowerment through the choreography, could influence the fact that the vigor-activity factor remained high after the performance, despite the fatigue. We also relate this result of the vigor-activity scale with the concept of “academic engagement,” a central construct to promote learning, performance, interest, enjoyment and psychological well-being among students (Medrano et al., 2015).

The third hypothesis is also confirmed in the sense that in our study, there are no significant differences with respect to the gender variable, in relation to the evaluation in Body expression and dance through a choreographic presentation. This result agrees with previous studies in which there are no differences between male and female university students, in relation to expression and dance practices (Gelpi et al., 2014; Romero-Martín et al., 2017). Details regarding adolescent attitudes toward dance, for example, and how they may vary with age and gender, are scarce (Sanderson, 2001). In previous studies with adolescents, the analyzes showed little change in attitudes between the ages of 11 and 16 and no interaction of age with gender. The absence of interaction between age and gender suggests cultural rather than school influences on attitudes. Similarly, Gaoa et al. (2014) examined whether childrens’ enjoyment of physical education varied as a function of learning activities. The results revealed that children reported significantly higher scores on enjoyment of interactive dance games than traditional games. In addition, girls showed greater enjoyment with the interactive dance games than the boys. However, no gender differences emerged in the enjoyment of traditional games. Ayyildiz and Gokyürek (2016) researched the levels of satisfaction in leisure of individuals who attend dance activities as a recreational activity in the dance course, in relation to the levels of satisfaction in leisure. There are no statistically significant differences between gender and the level of satisfaction with dance in leisure time.

On the other hand, there are studies that show differences between boys and girls in relation to the dance technique that is used. Amado et al. (2016), analyze motivation toward dance by comparing two teaching methodologies within the educational context. According to gender, the study revealed that girls felt more secure under the *Direct Instruction Technique* because they considered that they had the necessary resources to perform the movements successfully. On the other hand, under the Creative Inquiry Technique, they felt more ridiculous when they saw what they were doing and they believed that they were not doing it efficiently. The boys felt more secure under the Creative Inquiry Technique because they considered that they knew what they were doing and it was easier for them because they adapted their choreographies to their level of performance. Nevertheless, with the Direct Instruction Technique, they felt incompetent because, despite knowing the movements and repeating them, they believed that they were not efficient in their execution. And this suggests that teachers may need to apply a different treatment depending on gender.

In different studies, motor expression practices activated high values of positive emotions in students. The cooperative domain originated more intense positive emotions than the psychomotor domain. No significant differences were found in emotional intensity between men and women (Gelpi et al., 2014). Romero-Martín et al. (2017), conclude that the motor practices studied (psychomotor, expression practices and introjection practices) promote positive emotional experiences, highlighting the expression practices and games, which trigger the most intense positive emotions. Regarding gender, men registered more intense emotional values women in games and in negative and ambiguous emotions, while they had similar emotional behaviors in expression and introjection.

The internal logic of all cooperative games (such as our purpose of evaluation through choreography) requires that their protagonists interact, dialog, agree and speak to solve the group challenge that is presented to them. These processes associated with mutual help and empathy are the carriers of pleasant experiences of subjective and social well-being, as it has been shown in other studies based on traditional games, with and without competition (Lavega et al., 2011; Jaqueira et al., 2014), and also in non-competitive cooperative areas of motor expression (Sáez de Ocáriz et al., 2014), and of sensitive self-exploration or motor introjection (Rovira et al., 2014a, b).

The internal logic of this type of motor task provokes different behaviors in modifying moods, depending on whether cooperation is associated with competition or it is done in the absence of a final score (Muñoz Arroyave et al., 2014). Although this type of practice entails well-being and pleasant experiences that increase the vigor and activity of its protagonists, it can also increase or decrease other emotional factors such as stress, dejection, anger or fatigue depending on the cooperative challenge that is perceived and lived.

Likewise, our study would confirm the iceberg configuration studied by Morgan and Pollock (1977), who analyzed the differences in mood states between those who practiced and did not practice sports, and suggested that athletes present more positive mood profiles than non-athletes. The mood profile obtained by those who practiced sports presented the following characteristics: lower values than non-athletes in stress, depression, anger and fatigue, and high vàlues in vigor. Morgan (1980), who worked on his studies with male population, described this configuration as the “iceberg” profile. In our study, the vigor-activity scale, which shows only positive mental states, reflects that the scores are above other scales (stress-anxiety, depression-dejection, hostility-rage and fatigue-immobility).

We agree with Gelpi et al. (2014); Gómez-Carmona et al. (2019), when they conclude, in their study, that the motor practices of expression, characterized within the logic through the use, expression and communication of emotions, they constitute a family of motor practices that unleashes intense values of positive emotions.

### Limitations

Some limitations regarding the questionnaire itself should be noted. In Arce et al. (2000) the psychometric analyzes revealed that, relative to the original description of each mood state, some of adjectives have their original meaning and have been associated to a different mood factor. Another concurrent problem is the different usage of the number of items and their scale assignment (Morfeld et al., 2007). Possible variables such as, for example, the gender of the student body members of the different groups that showed their choreographies were not contemplated. The grouping by gender (men, women or mixed), according to Lavega et al. (2017), was not considered in our study as a variable to control. Therefore, we do not know, if the fact that they were homogeneous groups of men, homogeneous groups of women or mixed groups, could influence the moods before or after the performance of each choreography. Another possible variable is the level of security in the presentation of the choreography. It is possible that a choreography that is not overly rehearsed, or insecure, entails different states of mind than those of a rehearsed and thoroughly worked choreography, that the students want to show. Another consideration to be made regards the type of task that the evaluations entail. Even in the case of motor cooperation, a certain component of “challenge” or “competition” could be considered with oneself or in relation to the other groups that are also evaluated. It would be necessary trying to find out if the emotional experience of the students under evaluation with cooperative tasks of a socio-motor type goes through any experience of “competition.” On the other hand, although much of the research that uses the POMS questionnaire as a means for data collection instrument has been influenced, not all research follows the same procedures to collect information; some of them use the GES Games and Emotions Scale for study motor experiences (Lavega et al., 2018) in a unique way or in a complementary way, so caution should be exercised when comparing results.

## Conclusion

Cooperative games (among them, motor expression activities and also the use of choreography in some moments of the evaluation) constitute a domain or family of motor games of great interest for their contribution to the modification of the states of mind of the students. The POMS (Profile of Mood States) questionnaire allows information to be obtained on pre-performance and post-performance moods, as well as to compare them at two different times.

The mood states of the students before an assessment situation through motor choreography will be emotionally different from the state of mind once the performance is over. The practical evaluation of the subject through choreography modifies the states of mind of the students. It favors and increases the development of the subjective and collective well-being of the people who carry them out, which is why it is considered a positive element in the educational process.

We therefore noted differences in each of the choreographies, before and after each performance, and in the overall score awarded to both choreographies. The results indicate a decrease of the scores in all the scales, except for the fatigue-immobility scale, after the choreography. Because of this, we consider that the practice increases the emotional well-being of the students after performing their choreographies.

Although we anticipate a possible “training to the moods generated by the evaluation” in the performance of the choreographies, the stress-anxiety factor, the pre- and post-moods are similar in the first and second choreographies. We can highlight that the stress-anxiety scale is somewhat lower “before” the second choreography. Therefore, we can speak of a “training” of all students on this scale.

Regarding the moods before and after the scene will have similar values in men and women, we could not establish any differences between genders, although divergencies were found on the depression-dejection scale on this same variable, during the first phase and prior to performance.

The desire to show the choreography and the feeling of security prior to its performance contributed to a greater satisfaction after performing it. Motor expression activities, and specifically the evaluative moment of the same through representations, are manifested as an educational resource that favors and increases the development of subjective and collective well-being among the participants who carry them out. In this case, the cooperative nature of the activities mobilized positive emotional intensities, higher than other expressive psychomotor proposals.

This research confirms the relevant role of dance, as well as the emotional repercussions of its evaluation, for university students. The evaluation of the emotional state of fellow spectators is pending, before and after esthetically perceiving the choreographies of their classmates (Cross et al., 2011), that are probably linked to positive emotions. That is why educating the expressive dimension of the body through dances, and the values associated with their practice, should be one of the objectives of training programs in Physical Education. The findings of this research provide relevant information for physical education professionals in relation to the effects of evaluating dance and body expression practices.

It is clear how important it is for Physical Education teachers to know the emotional experience generated by evaluations according to the type of tasks that they introduce to their students, thus being able to guide their teaching-learning process. To promote a correct emotional education from a physical education area, it is necessary to teach students to recognize, control and understand their own emotions in different motor situations. From this point of view, it is essential that future teachers in this subject experience for themselves the emotions that they provoke through different forms of evaluation that they suggest to their students in the future provoke, in order to recognize them and be able to work on them with their students.

## Data Availability Statement

The raw data supporting the conclusions of this article will be made available by the authors, without undue reservation.

## Ethics Statement

Written informed consent was obtained from the individual(s) for the publication of any potentially identifiable images or data included in this article.

## Author Contributions

MM involved in the design of study, data collection, data analysis, and manuscript writing. SG contributed to the data collection and field work, data analysis, and manuscript writing. LS assisted data acquisition. AA contributed to data analysis, run analysis of results, and manuscript writing. EF contributed to manuscript writing and review of the document. All authors contributed to the article and approved the submitted version.

## Conflict of Interest

The authors declare that the research was conducted in the absence of any commercial or financial relationships that could be construed as a potential conflict of interest.

## Footnotes

1 https://tanzfonds.de/en/home/

2 http://terpsichore-project.eu/
